# Motivational Climate Is Associated with Use of Video Games and Violence in Schoolchildren: A Structural Equation Model According to Healthy Behaviors

**DOI:** 10.3390/ijerph17041272

**Published:** 2020-02-17

**Authors:** Manuel Castro-Sánchez, Amador Jesús Lara-Sánchez, Eduardo García-Mármol, Ramón Chacón-Cuberos

**Affiliations:** 1 Department of Didactics of Musical, Plastic and Corporal Expression, University of Granada, 18071 Granada, Spain; manuelcs@ugr.es; 2 Department of Didactics of Musical, Plastic and Corporal Expression, University of Jaén, 23071 Jaén, Spain; alara@ujaen.es; 3 Department of Physical Education. University of Granada, 18071 Granada, Spain; eduardogarcia@ugr.es; 4 Department of Research Methods and Diagnosis in Education, University of Granada, 18071 Granada, Spain

**Keywords:** motivation, bullying, new technologies, leisure time

## Abstract

The aim of this cross-sectional study was to develop an explanatory model of motivational climate, problematic use of videogames, violent behaviour and victimisation in schoolchildren. The sample included 734 children aged between 10 and 12 years of age from the province of Granada (Spain). A multi-group structural equation model was used, with an excellent fit (CFI = 0.964; NFI = 0.954; IFI = 0.964; RMSEA = 0.048). The results showed a positive relationship between the problematic use of video games, victimisation and violent behaviors, associating negatively with the task climate. Likewise, the task-oriented motivational climate was indirectly related to victimisation situations and violent behavior, while the ego climate did so positively with special emphasis on children who did not perform physical activity. As a main conclusion it is shown that adherence to the practice of physical activity, and particularly within a task-oriented motivational climate, can act as a protective factor against the problematic use of video games.

## 1. Introduction

The rapid progress of technological development during the last decade has introduced a multitude of devices into the average home and society in general. These devices have led to an expansion of the entertainment industry, which now offers new ways to spend time, replacing sports and physical activities with predominantly sedentary pursuits [1]. As a result, society is facing a problem characterised by a decrease in healthy active pursuits and an increase in sedentary pursuits, which in some cases means the abuse of videogames and other technological media, which can gravely affect the youngest members of society [2].

Shi et al. [3] suggested that excessive use of videogames during school years is a risk factor associated with physical, social and cognitive health problems. Van Rooij et al. [4] identified the main potential harms as being eye problems and hormonal changes, in addition to cognitive disorders that are associated with stress and depression. Possibly the most noticeable defects are to the social and emotional skills of the youngest users, resulting in damaged relationships. These problems arise due to a lack of parental control in the use of this type of technology in large part because of a lack of knowledge of the negative effects [5].

Numerous studies have analysed the relationship between pathological videogame use, behavioural problems and a general deterioration in health included increased obesity and excess weight due to the increasingly sedentary nature of leisure pursuits [6,7]. Lau et al. [8] revealed a direct relationship between increased videogame usage, increased aggressiveness and victimisation. These behaviours can be explained by the content of videogames (in the case of videogames which are predominantly violent) and ultimately result in a deterioration in social relations [9]. The abuse of videogames is therefore a risk factor in the initial occurrence and/or rise in aggressive behaviour in youngsters [10]. Videogames expose users to a repertoire of negative conduct that is often easy to imitate. Young users who have not yet fully developed correct social skills are particularly susceptible to copying behaviours experienced in this way [11].

Prolonged use of violent videogames is associated with cognitive processes that favour violence and produce a desensitisation towards violent behaviour. In addition, the role of the aggressor as the protagonist in videogames result in users who no longer fear violent situations, identify with the aggressor rather than the victim and increasingly tolerate violent behaviour [12]. Furthermore, the abuse of smartphones, social media, and videogames is directly associated with victimisation, making subjects more susceptible to suffering violence due to the loss of social skills caused by inadequate assimilation with the peer group [13].

Physical activity is promoted as a healthy substitute for sedentary habits and the negative behaviour associated with videogames. Physical activity is a building block that is capable of encouraging further positive behaviour and of counteracting the damaging conduct associated with sedentary habits [14]. Research into the motivational processes that promote physical activity in young people is fundamentally important because perceptions developing during school age will largely determine whether a child will continue to practice sports regularly into adolescence and adulthood, or instead adopt sedentary pastimes that are related with numerous health problems [15]. Clark et al. [16] analysed students’ motivations towards physical activity as positive types of motivation are fundamental in initiating and maintaining physical activity engagement, and preventing the appearance of sedentary habits.

Achievement goal theory is one of the most frequently applied theories when studying motivation in the context of physical activity and sports. According to achievement goal theory, an individual’s motivation towards engaging in sport or physical activity depends on the types of goals they set for themselves. These goals are called achievement goals [17,18].

According to achievement goal theory there are two different types of motivational orientations towards engagement in any behaviour, in this case we will discuss physical activity and sports. The two orientations are not mutually exclusive, but are complementary, in that individuals tend to exhibit different levels of both. Namely, they are task (mastery) goals and ego (performance) goals, with task goals typically dominating. Task-oriented goals are predominantly self-determined and are characterised by a hedonistic involvement in which the individual seeks personal satisfaction or enjoyment. This is associated with a greater affinity with the sport of physical activity practised by the individual. On the other hand, an ego-oriented climate is less self-determined, in that the individual is motivated predominantly by outcome achievement and external rewards. This is associated with greater intra-team rivalry and a lower likelihood of continuing to practise sports [19,20].

The aim of this study was to develop an explanatory model of the problematic use of videogames, motivational climate, violent behaviour and victimisation in schoolchildren. In addition, the second aim of this paper was to analyse the existing relationship between these variables according to a participant’s engagement with physical activity using multi-group analysis.

## 2. Materials and Methods

### 2.1. Design and Participants

A descriptive, cross-sectional study was conducted with a sample of 734 students (45.2% boys and 54.8% girls). Participants were aged between 10 and 12 years old (M = 10.88 years; SD = 0.69) and were enrolled in the fifth and sixth year of primary school in the city of Granada. Convenience sampling was used, with the proviso that only students in the third cycle of primary education would be invited. Participants were recruited from 11 education centres in Granada. Participation was entirely voluntarily. Individual monitoring assured that no data was duplicated.

### 2.2. Variables and Instruments

The analysis of the problematic use of video games was done using the Videogame Experience Questionnaire (VEQ) adapted into Spanish by Chamarro et al. [21]. The questionnaire comprises 17 negatively framed items, valued on a 4-point Likert scale on which 1 = Never; 2 = Sometimes; 3 = Almost Always; 4 = Always. Items were summed to establish an overall score describing participant behaviour relating to the use of videogames. Data were divided between tertiles which categorised behaviour as: “No Problems”, “potential problems”, and “severe problems”. Cronbach’s alpha for the original questionnaire reported by Chamarro et al. [21] was α = 0.87, and in the present study was α = 0.91.

The Multidimensional Peer-Victimisation Scale (MPVS) was developed by Mynard and Joseph [22] and adapted into Spanish by Cava et al. [23]. The scale comprises 20 items measured on a 4-point Likert scale (1= Never; 4 = Always) which pertain to three types of victimisation: physical victimisation, verbal victimisation and relational victimisation. The original Mynard and Joseph [22] study obtained an internal consistency (Cronbach’s alpha) of α = 0.77. The present study obtained a superior coefficient of α = 0.93. Individual sub-scales scored as follows: relational victimisation (α = 0.88), physical victimisation (α = 0.86), and verbal victimisation (α = 0.84). Similar scores were identified in a study conducted by Povedano et al. [24].

Violent behaviour was assessed through the School Aggressiveness Scale (SAC), originally developed by Little et al. [25] and adapted in Spanish by the Lisis Group [26]. This scale has been used in similar studies such as by Cava et al. [23] and Musitu et al. [27]. The scale is divided into two categories: overt or direct aggressiveness (generated in a face-to-face meeting where the aggressor can be identified by the victim); and indirect or relational aggressiveness (when the aggressor is more or less anonymous). Each category is subdivided into three subscales: simple, reactive and instrumental. The scale consists of 25 items and responses are given on a 4-point Likert scale ranging from 1 (never) to 4 (always). The present study obtained a Cronbach’s alpha score of α = 0.856 for overt aggressiveness and α = 0.742 for relational aggressiveness, which is similar to values reported by Musitu et al. [27] (α = 0.088 and α = 0.081, respectively).

Motivation was measured using the Perceived Motivational Climate in Sports Questionnaire (PMCSQ-2) [28], specifically the Spanish version adapted by González-Cutre et al. [29] of the original scale done by Newton, Duda, and Yin [28]. This tool comprises 33 items measured on a 5-point Likert scale ranging from 1 = Totally disagree, to 5 = Totally agree. The questionnaire defines two categories, each with three subscales: the first category of subscales relates to a task climate and are named cooperative learning, effort/improvement and important role. The second category of subscales relate to an ego-involving climate and are named punishment of mistakes, unequal recognition and intra-team rivalry. Internal consistency (Cronbach’s alpha) of the Spanish version of the instrument obtained by González-Cutre et al. [29] was α = 0.90 for the ego dimension. The internal consistency of each ego subscale was as follows: punishment of mistakes (α = 0.77), unequal recognition (α = 0.87) and intra-team rivalry (α = 0.61). The present research obtained an internal consistency score of α = 0.89 for the ego-oriented climate dimensions. The scores obtained in the present study for the ego sub-scales were as follows: punishment of mistakes (α = 0.92), unequal recognition (α = 0.91) and intra-team rivalry (α = 0.68). The internal consistency of the task-oriented climate dimensions was α = 0.93. Scores obtained for the task sub-scales were as follows: cooperative learning (α = 0.83), effort/improvement (α = 0.84), and important role (α = 0.86).

To record the level of physical activity practice, participants responded to an ad hoc questionnaire reporting whether they habitually engaged in more than three hours a week of sports outside of school hours. Responses were given as a yes or no response.

### 2.3. Procedure

Permission to approach and invite schools to participate in the study was obtained from the Granada University Science Faculty and the Andalusian Government Department of Education. The school’s directors were informed of the nature of the study and a request for student participation was made. Informed consent was obtained from legal guardians as participants were under 18 years old.

Participants were informed that all information gathered was to be used solely for scientific purposes. Study personnel were present during the collection of all study data in order to resolve problems or address doubts relating to study processes. Teachers, support staff and others were thanked for their collaboration and informed that they would be sent a report on the outcomes of the study.

Of the data collected, 52 questionnaires were discarded due to incorrect completion. Ethical guidelines laid out in the Declaration of Helsinki (World Medical Association) concerning research projects (Law 223/2004 of 6 February) were followed, as was national legislation on clinical trials (relating to biomedicine Law 14/2007 of 3 July) and law regarding confidentiality (Law 15/1999 of 13 December).

### 2.4. Data Analysis

IBM SPSS^®^ version 22.0 (IBM Corp, Armonk, NY, USA) for Windows was used for the basic descriptive analysis. The IBM AMOS^®^ 23 programme (IBM Corp, Armonk, NY, USA) was used to analyse the relevant constructs included in a structural model. Once the theoretical model was constructed, the paths were analysed according to their relation to the matrix using multi-group analysis, grouping participants according to engagement with physical activity (with level of physical activity being entered as grouping variable). Two different structural models were configured with the aim of verifying whether the relations between variables varied according to whether students were physically active or not.

**Figure 1:**
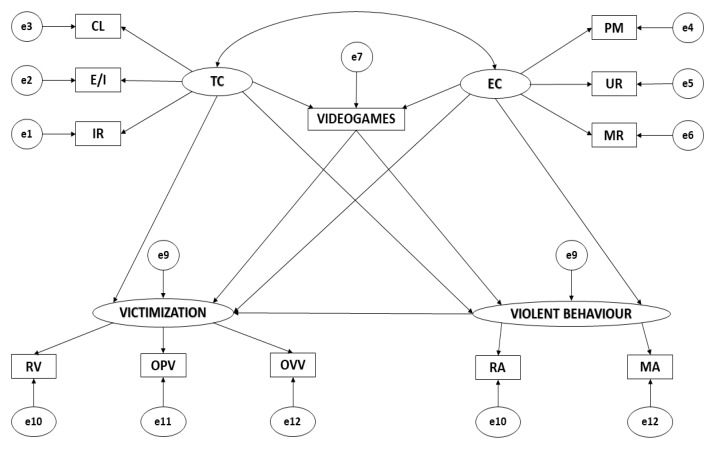
Theoretical Model. Note: TC, task climate; CL, cooperative learning; E/I, effort/improvement; IR, important role; EC, ego climate; ITR, intra-team rivalry; PM, punishment of mistakes; UR, unequal recognition; VIDEOGAMES, use of videogames; VICTIMISATION, victimisation; RV, relational victimisation; OPV, overt physical victimisation; OVV, overt verbal victimisation; VIOLENT BEHAVIOUR, violent behaviour; MA, manifest aggressiveness; RA, relational aggressiveness.

Path diagrams included 12 observable variables and 12 latent variables which determined the indicators (Figure 1). The causal explanations for the latent variables were formulated based on the observed relations between indicators and the reliability of the measurements was evaluated. Measurement error was directly controlled within the included observable variables. Single-direction arrows show the direction of influence between latent and observable variables and are interpreted as multivariate regression coefficients. Bi-directional arrows show the relationships between latent variables and the regression coefficients.

Task-oriented motivational climate (TC) and ego-oriented motivational climate (EC) act as exogeneous variables with each being inferred from three indicators. For the task-oriented climate, the indicators are: important role (IR), effort/improvement (E/I) and cooperative learning (CL). For the EC, the indicators are: punishment of mistakes (PM), unequal recognition (UR) and intra-team rivalry (ITR). The use of videogames (VIDEOGAMES) acts as an endogenous variable, acted upon by task-oriented climate (TC) and ego-oriented climate (EC), and victimisation (VICTIMISATION). Victimisation also acts as an endogenous variable, acted upon by the use of videogames (VIDEOGAMES), task-oriented climate (TC), and ego-oriented climate (EC).

Model fit was tested to verify compatibility with the empirical data. Goodness of fit evaluation [30] was conducted. Chi-squared test analysis produced insignificant *p*-values, suggesting good fit. Comparative fit index (CFI) values above 0.90 suggest acceptable fit and values above 0.95 suggest excellent fit. Normed fit index (NFI) values above 0.90 are acceptable. Index of adjusted increment (IFI) values above 0.90 are acceptable and values above 0.95 are excellent. Finally, Root Mean Square Error of Approximation (RMSEA) values of less than 0.08 are acceptable and values less than 0.05 are excellent.

## 3. Results

The structural equation model developed, controlling for sex, showed good fit for all evaluation indexes. Chi-squared analysis produced a significant p value (χ2 = 395.325; DF = 90; *p* < 0.001). However, this index cannot be interpreted in the standard manner, as sample size poses a problem to its sensitivity [30]. For this reason, other standardised fit indexes were used which are less sensitive to sample size. CFI was 0.964, NFI was 0.954, IFI was 0.964 and RMSEA was 0.048. All of these values describe excellent fit to the data.

**Figure 2:**
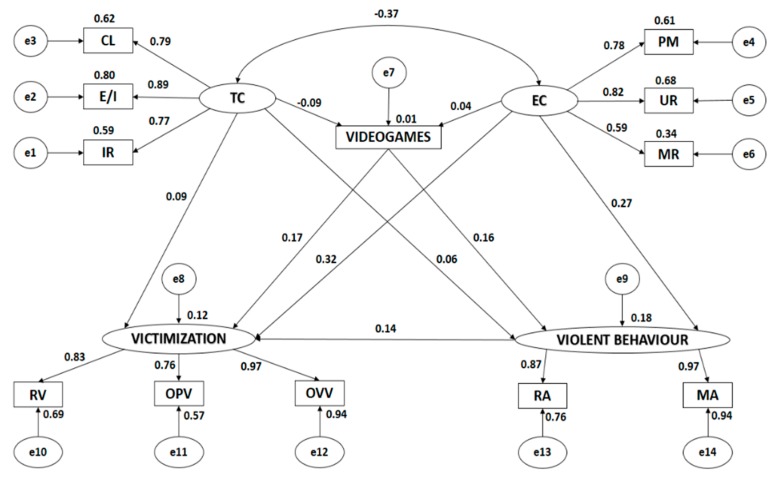
Structural equation model for physical activity. Note: TC, task climate; CL, cooperative learning; E/I, effort/improvement; IR, important role; EC, ego climate; ITR, intra-team rivalry; PM, punishment of mistakes; UR, unequal recognition; VIDEOGAMES, use of videogames; VICTIMISATION, victimisation; RV, relational victimisation; OPV, overt physical victimisation; OVV, overt verbal victimisation; VIOLENT BEHAVIOUR, violent behaviour; MA, manifest aggressiveness; RA, relational aggressiveness.

Both Figure 2 and Table 1 show the estimated values for the model parameters of the structural model for students who habitually practice physical activity. Values should significantly differ from zero and negative variables are undesirable.

All categories of motivational climate and its dimensions were positively and directly associated (*p* < 0.005). A task-oriented climate was negatively and indirectly associated with an ego-oriented climate (*p* < 0.005, *r* = −0.365). Victimisation was significantly and positively associated with all of its indicators (*p* < 0.005). This was also the case for violent behaviour and its indicators.

The indicators of influence for each latent variable were all positively and directly associated (*p* < 0.005). A task-oriented climate was most strongly associated with the indicator describing effort/improvement (*r* = 0.894), followed by cooperative learning (*r* = 0.785) and important role (*r* = 0.766). An ego-oriented climate was most strongly associated with the indicator describing unequal recognition (*r* = 0.823), followed by punishment of mistakes (*r* = 0.779) and intra-team rivalry (r = 0.585). Victimisation was most strongly associated with verbally manifest victimisation (*r* = 0.968), followed by relational victimisation (*r* = 0.829) and manifest physical victimisation (*r* = 0.756). Violent behaviour was most strongly associated with manifest aggressiveness (*r* = 0.969), followed by relational aggressiveness (*r* = 0.871).

Similarly, a positive indirect association (*p* < 0.008) was observed between a task-oriented climate and victimisation (*r* = −0.089). Negative and indirect associations were observed between a task-oriented climate and videogame use (*r* = −0.095, *p* = 0.005), and a task-oriented climate and violent behaviour (*p* = 0.012, *r* = −0.082). With regards to an ego-oriented climate, this variable was positively and directly associated with victimisation (*p* < 0.005, *r* = 0.317) and with violent behaviour (*r* = 0.272), with the strength of association being moderate. An ego-oriented climate was not significantly associated with videogame use (*p* = 0.215).

Videogame use was positively and directly associated with victimisation (*p* < 0.005, *r* = 0.170) and violent behaviour (*r* = 0.159; *p* < 0.005), with the strength of association being low. Finally, a direct positive relationship was observed between victimisation and violent conduct (*r* = 0.137, *p* < 0.005).

**Figure 3:**
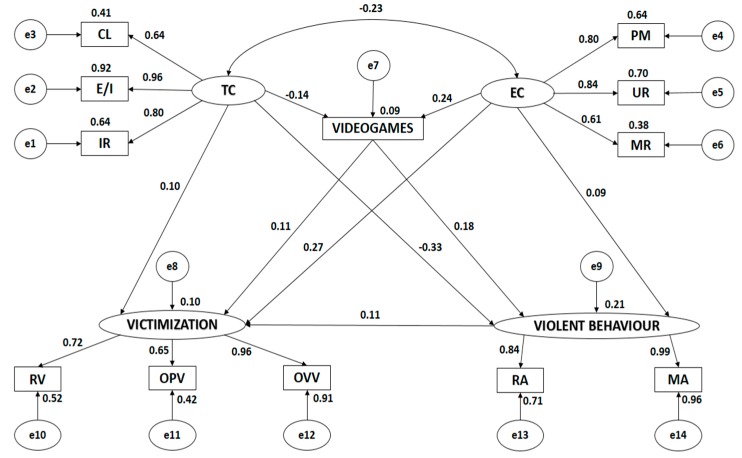
Structural equation model in sedentary subjects. Note: TC, task climate; CL, cooperative learning; E/I, effort/improvement; IR, important role; EC, ego climate; ITR, intra-team rivalry; PM, punishment of mistakes; UR, unequal recognition; VIDEOGAMES, use of videogames; VICTIMISATION, victimisation; RV, relational victimisation; OPV, overt physical victimisation; OVV, overt verbal victimisation; VIOLENT BEHAVIOUR, violent behaviour; MA, manifest aggressiveness; RA, relational aggressiveness.

Both Figure 3 and Table 2 show the estimated values of the structural model parameters for schoolchildren who do not habitually practise physical activity. Value should significantly differ from zero and negative variables are undesirable.

All categories of motivational climate were positively and directly associated with its dimensions (*p* < 0.005). A task-oriented climate was negatively and indirectly related with an ego-oriented climate (*p* < 0.005, *r* = −0.203). Victimisation was positively and directly associated with all of its indicators, as was violent behaviour (*p* < 0.005).

Indicators of all latent variables were positively and directly associated (*p* < 0.005). A task-oriented climate was most strongly associated with the indicator describing effort/improvement (*r* = 0.957), followed by important role (*r* = 0.800) and cooperative learning (*r* = 0.638). An ego-oriented climate was most strongly associated with the indicator describing unequal recognition (*r* = 0.837), followed by punishment of mistakes (*r* = 0.798) and intra-team rivalry (*r* = 0.613). Victimisation was most strongly associated with the indicator describing overt verbal victimisation (*r* = 0.956), followed by relational victimisation (*r* = 0.720) and overt physical victimisation (*r* = 0.651). Violent behaviour was most strongly associated with manifest aggressiveness (*r* = −0.990) followed by relational aggressiveness (*r* = 0.843).

Similarly, a task-oriented climate was negatively and indirectly associated with videogame use (*p* < 0.024, *r* = −0.142) and violent behaviour (*p* < 0.005, *r* = −0.326). No significant relationship was identified between a task-oriented climate and victimisation (p = 0.112). An ego-oriented climate was directly and positively associated with victimisation (*p* < 0.005, *r* = 0.275) and use of videogames (*r* = 0.242). An ego-oriented climate was not significantly associated with violent behaviour (*p* = 0.179).

Videogame use was positively and directly associated with violent behaviour (*p* = 0.002, *r* = 0.180), with the strength of association being weak. No significant association was identified between videogame use and victimisation (*p* = 0.092), nor between victimisation and violent behaviour (*p* = 0.077).

## 4. Discussion

The aim of this study was to develop multi-group SEMs, which compared the associations between motivational climate toward sport, victimisation, violent behaviour, and the problematic use of videogames. The path models developed demonstrated excellent fit, suggesting that they validly explained the relationships between the variables measured in the present sample of schoolchildren. Fit indices for this model, which also incorporated information on the individual’s engagement in physical activity, were similar to those identified in several studies previously conducted in both national and international contexts [11,31,32,33,34]. Specifically, the present study seeks to take one more step than in the study conducted by Castro-Sánchez et al. [32]. Now, not only is the aim to analyse the relationships between violence, videogame use and motivational climate in school children, but it is also intended to analyse the moderating effect of healthy habits through a multi-group analysis with structural equations.

Concerning the motivational climate perceived towards sport, the SEM results produced significant inverse relationships between a task-oriented climate and an ego-oriented climate for children who habitually practiced physical activity and those who did not, though this relationship was strongest in those children who practised sports regularly. These results indicate that children who more strongly perceive a task-oriented motivational climate have lower perceptions of an ego-oriented climate [18,35]. Individuals with stronger task orientations tend to prioritise personal growth and are motivated intrinsically. On the other hand, individuals with stronger ego orientations tend to prioritise external rewards and are motivated more by external factors [19]. This relationship was more evident in schoolchildren who regularly engaged in physical activity than in schoolchildren who did not engage regularly in physical activity. Engagement in physical activity has been shown to promote the acquisition of conducts related to intrinsic motivational factors centred on personal growth, cooperation and the development of healthy habits [36].

With regards to the individual indicators of task climate, the indicator that asserted most influence on both groups (inactive/physically active) was effort/improvement. In physically active children, the least influential indicator was important role. In inactive children, the least influential indicator was cooperative learning. These results can be explained by the fact that regular participants in physical activity give greater importance to the effort and cooperation of team sports, whereas those who do not regularly participate value demonstrating ability and having an important individual role [37,38].

With regard to ego-oriented climate, its indicators followed a similar pattern in both physically active and physically inactive schoolchildren, despite being slightly more evident in physically inactive schoolchildren. The most influential indicator was unequal recognition, followed by punishment of mistakes and intra-team rivalry [39,40].

Concerning victimisation in schoolchildren, relational victimisation and manifest physical victimisation were revealed to be the two indicators with greatest influence in both active schoolchildren and inactive schoolchildren, although the strength of correlations were higher among schoolchildren who habitually engaged in physical activity. This outcome is expected as engagement in sport exposes the individual to a multitude of opportunities for cooperation, competition and to experience conflict, all of which improve social relations. In addition, the increased physical contact between participants means there is a greater prospect of suffering relational and physical violence [41,42].

The most common aggressive behaviour identified in this sample of schoolchildren was manifest aggressiveness, with a greater incidence in inactive schoolchildren. The weakest indicator was relational violence, with a stronger relationship evident in those who engaged in sport habitually. Manifest violence is a less complex behaviour that is typically exhibited among primary schoolchildren, while relational violence is more commonly seen among older students [43]. Those who habitually practice sport are more accustomed to experience relational violence than those rarely engage in sport due to the social interactions and conflicts that arise [44].

There was a direct positive relationship between motivational climate and victimisation, with the strongest correlation being for an ego-oriented climate among schoolchildren who habitually engaged in sport. The relationship between an ego-oriented climate and victimisation can be explained by the more egocentric character, which is often exhibited among ego-oriented schoolchildren, who tend to be more competitive and often view their peers as rivals. This produces confrontations that can give rise to violence between companions [45,46].

A negative indirect association was identified between a task-oriented climate and problematic use of videogames, while a direct and positive association existed between an ego-oriented climate and problematic use of videogames in physically inactive schoolchildren. This relationship suggests that a task-oriented climate encourages healthy habits and greater engagement in physical activity, while an ego-oriented climate is associated with maladaptive conduct such as the abusive use of videogames and other screen-based activities. It is, therefore, possible that physically inactive schoolchildren with higher ego-oriented climate perceptions will experience more problematic use of videogames than those who are oriented more towards perceptions of a task climate [47,48].

There was a negative relationship between a task-oriented climate and violent behaviour in schoolchildren who engaged rarely or never in physical activity. A positive relationship existed between an ego-oriented climate and violent behaviour in schoolchildren who habitually engaged in physical activity. These findings corroborate those reported in previous studies [49,50], with direct relationships reported between an ego-oriented climate, aggressiveness and violent behaviour, and a task-oriented climate being related with lower levels of aggression [51]. An ego-oriented motivational climate is characterised by greater levels of rivalry between members of a group, greater competitiveness and maladaptive behaviour, which can lead to an increase in violent behaviour. A task-oriented climate is related with healthier habits including cooperation, effort and personal growth, behaviours which breed lower levels of aggressiveness [52,53].

The present analysis showed a positive and direct relationship between victimisation and violent behaviour with problematic videogame use. Those who abuse videogames are more prone to becoming the victims of stalkers, although the relationship between use of videogames and victimisation is only present in schoolchildren who regularly engage in sport. The relationship between use of videogames and violent behaviour was evident in both active and inactive schoolchildren, although the strongest correlation was among physically inactive schoolchildren. These findings support those generally found in previous studies of the same topic [5,54]. An explanation for these findings is that children exposed to videogames with violent content tend to experience feelings of victimisation, which can produce violent reactions. Many videogames contain violent material and it is challenging for many parents to control the media to which their children are exposed. Abusive use of videogames has also been shown to be a risk factor of increases in bullying at school [55,56].

Finally, a positive direct relationship was found between victimisation and violent behaviour in schoolchildren who regularly engaged in physical activity. Individuals who regularly experience negative feelings of receiving cruel or unfair treatment are likely to retaliate aggressively due to continual exposure to these sentiments [57]. Participation in physical activity can be a protective factor against this response as participants become accustomed to negative physical and emotional contact and learn to manage their feelings and deal with ideas of physical violence [58,59].

A main limitation of the present study is that it was cross-sectional, which precludes causal conclusions from being made. We therefore recommend the development and examination of intervention programmes aimed at encouraging regular engagement in physical activity as a substitute for sedentary pursuits including the use of videogames. As the main practical implication derived from the results found in this research, it is essential to promote a positive motivational climate within the classroom which promotes task-oriented processes over ego-oriented processes, leading to greater benefits and reduced maladaptive behaviour.

## 5. Conclusions

The main conclusions of the present study are that an ego-oriented climate is positively related to victimisation, whereas victimisation and violent behaviour are negatively related to a task-oriented climate. These relationships are also stronger among physically inactive schoolchildren. Furthermore, the negative relationship between a task-oriented climate and the use of videogames was also stronger within physical inactive schoolchildren. Associations relating to an ego-related climate and videogame use were stronger among children who engaged in less than 3 hours’ peer week of physical activity. Videogame use was positively related with violent behaviour in both groups of schoolchildren, though associations were stronger within physically inactive schoolchildren. It can therefore be established that a task-oriented climate could act as a protective factor against the problematic use of video games and victimisation. Furthermore, following an inactive lifestyle is related to maladaptive behaviours such as the problematic use of video games and violent behaviours.

## Figures and Tables

**Table 1:** Structural model for physical activity.

Relation between Variables			R.W.				S.R.W.
			EST.	E.E.	C.R.	P	EST.
VIDEOGAMES	←	TC	−1.039	0.373	−2.787	***	−0.095
VIDEOGAMES	←	EC	0.456	0.368	1.239	.215	0.044
VICTIMISATION	←	EC	0.508	0.057	8.849	***	0.317
VICTIMISATION	←	TC	0.150	0.057	2.653	*	0.089
VICTIMISATION	←	VIDEOGAMES	0.026	0.004	5.943	***	0.170
VIOLENT BEHAVIOUR	←	EC	0.166	0.022	7.473	***	0.272
VIOLENT BEHAVIOUR	←	TC	−0.053	0.021	−2.517	*	−0.082
VIOLENT BEHAVIOUR	←	VIDEOGAMES	0.009	0.002	5.657	***	0.159
VIOLENT BEHAVIOUR	←	VICTIMISATION	0.052	0.012	4.485	***	0.137
IR	←	TC	1.000	-	-	-	0.766
E/I	←	TC	1.195	0.042	28.316	***	0.894
CL	←	TC	1.035	0.038	26.919	***	0.785
PM	←	EC	1.000	-	-	-	0.779
UR	←	EC	0.502	0.023	22.105	***	0.823
ITR	←	EC	0.840	0.046	18.294	***	0.585
OVV	←	VICTIMISATION	1.000	-	-	-	0.968
OPV	←	VICTIMISATION	0.594	0.018	32.235	***	0.756
RV	←	VICTIMISATION	0.788	0.021	37.052	***	0.829
MA	←	VIOLENT BEHAVIOUR	1.000	-	-	-	0.969
RA	←	VIOLENT BEHAVIOUR	0.933	0.041	22.745	***	0.871
EC	↔	TC	−0.178	0.019	−9.491	***	−0.365

Note 1: TC, task climate; CL, cooperative learning; E/I, effort/improvement; IR, important role; EC, ego climate; ITR, intra-team rivalry; PM, punishment of mistakes; UR, unequal recognition; VIDEOGAMES, use of videogames; VICTIMISATION, victimisation; RV, relational victimisation; OPV, overt physical victimisation; OVV, overt verbal victimisation; VIOLENT BEHAVIOUR, violent behaviour; MA, manifest aggressiveness; RA, relational aggressiveness. Note 2: R.W., regression weight; S.R.W., standardised regression weight; E.E., estimation error; C.R., critical ratio. Note 3: *p* < 0.05 *; *p* < 0.01 **; *p* < 0.005 ***.

**Table 2:** Structural model in sedentary subjects.

Relations between Variables			R.W.				S.R.W.
			EST	E.E.	C.R.	P	EST
VIDEOGAMES	←	TC	−1.458	0.645	−2.260	*	−0.142
VIDEOGAMES	←	EC	2.623	0.729	3.597	***	0.242
VICTIMISATION	←	EC	0.377	0.100	3.761	***	0.275
VICTIMISATION	←	TC	0.137	0.086	1.590	0.112	0.105
VICTIMISATION	←	VIDEOGAMES	0.014	0.008	1.686	0.092	0.108
VIOLENT BEHAVIOUR	←	EC	0.057	0.043	1.342	0.179	0.090
VIOLENT BEHAVIOUR	←	TC	−0.197	0.037	−5.352	***	−0.326
VIOLENT BEHAVIOUR	←	VIDEOGAMES	0.011	0.003	3.117	***	0.180
VIOLENT BEHAVIOUR	←	VICTIMISATION	0.049	0.028	1.768	0.077	0.107
IR	←	TC	1.000	-	-	-	0.800
E/I	←	TC	1.209	0.085	14.233	***	0.957
CL	←	TC	0.847	0.076	11.126	***	0.638
PM	←	EC	1.000	-	-	-	0.798
UR	←	EC	0.497	0.045	11.054	***	0.837
MR	←	EC	1.018	0.108	9.415	***	0.613
OVV	←	VICTIMISATION	1.000	-	-	-	0.956
OPV	←	VICTIMISATION	0.596	0.058	10.268	***	0.651
RV	←	VICTIMISATION	0.660	0.059	11.180	***	0.720
MA	←	VIOLENT BEHAVIOUR	1.000	-	-	-	0.990
RA	←	VIOLENT BEHAVIOUR	0.884	0.076	11.643	***	0.843
EC	↔	TC	−0.123	0.039	−3.159	***	−0.230

Note 1: TC, task climate; CL, cooperative learning; E/I, effort/improvement; IR, important role; EC, ego climate; ITR, intra-team rivalry; PM, punishment of mistakes; UR, unequal recognition; VIDEOGAMES, use of videogames; VICTIMISATION, victimisation; RV, relational victimisation; OPV, overt physical victimisation; OVV, overt verbal victimisation; VIOLENT BEHAVIOUR, violent behaviour; MA, manifest aggressiveness; RA, relational aggressiveness. Note 2: R.W., regression weight; S.R.W., standardised regression weight; E.E., Estimation error; C.R., critical ratio. Note 3: *p* < 0.05 *; *p* < 0.01 **; *p* < 0.005 ***.

## References

[1] Anderson E.L., Steen E., Stavropoulos V. (2017). Internet use and problematic internet use: A systematic review of longitudinal research trends in adolescence and emergent adulthood. Int. J. Adol. Youth.

[2] Krossbakken E., Torsheim T., Mentzoni R.A., King D.L., Bjorvatn B., Lorvik I.M., Pallesen S. (2018). The effectiveness of a parental guide for prevention of problematic video gaming in children: A public health randomized controlled intervention study. J. Behav. Addict..

[3] Shi J., Boak A., Mann R., Turner N.E. (2018). Adolescent problem video gaming in urban and non-urban regions. Int. J. Ment. Health Addict..

[4] Van Rooij A.J., Schoenmakers T.M., Van de Mheen D. (2017). Clinical validation of the C-VAT 2.0 assessment tool for gaming disorder: A sensitivity analysis of the proposed DSM-5 criteria and the clinical characteristics of young patients with ‘video game addiction’. Addict. Behav..

[5] Verheijen G.P., Burk W.J., Stoltz S.E., van den Berg Y.H., Cillessen A.H. (2018). Friendly fire: Longitudinal effects of exposure to violent video games on aggressive behavior in adolescent friendship dyads. Aggress. Behav..

[6] Carras M.C., Van Rooij A.J., Van de Mheen D., Musci R., Xue Q.L., Mendelson T. (2017). Video gaming in a hyperconnected world: A cross-sectional study of heavy gaming, problematic gaming symptoms, and online socializing in adolescents. Comput. Hum. Behav..

[7] Hinkley T., Salmon J.O., Okely A.D., Crawford D., Hesketh K. (2012). Preschoolers’ physical activity, screen time, and compliance with recommendations. Med. Sci. Sport Exerc..

[8] Lau C., Stewart S.L., Sarmiento C., Saklofske D.H., Tremblay P.F. (2018). Who is at risk for problematic video gaming? Risk factors in problematic video gaming in clinically referred canadian children and Adolescents. Mult. Technol. Interact..

[9] Greitemeyer T. (2018). The spreading impact of playing violent video games on aggression. Comput. Hum. Behav..

[10] Szycik G.R., Mohammadi B., Hake M., Kneer J., Samii A., Münte T.F., Te Wildt B.T. (2017). Excessive users of violent video games do not show emotional desensitization: An fMRI study. Brain Imaging Behav..

[11] Adachi P.J., Willoughby T. (2017). The link between playing video games and positive youth outcomes. Child Dev. Perspect..

[12] Kimmig A.C., Andringa G., Derntl B. (2018). Potential adverse effects of violent video gaming: Interpersonal-affective traits are rather impaired than disinhibition in young adults. Front. Psychol..

[13] Cui J., Lee C., Bax T. (2018). A comparison of ‘psychosocially problematic gaming’ among middle and high school students in China and South Korea. Comput. Hum. Behav..

[14] Lewis B.A., Napolitano M.A., Buman M.P., Williams D.M., Nigg C.R. (2017). Future directions in physical activity intervention research: Expanding our focus to sedentary behaviors, technology, and dissemination. J. Behav. Med..

[15] Gillison F.B., Standage M., Cumming S.P., Zakrzewski-Fruer J., Rouse P.C., Katzmarzyk P.T. (2017). Does parental support moderate the effect of children’s motivation and self-efficacy on physical activity and sedentary behaviour?. Psychol. Sport Exerc..

[16] Clark H., Camiré M., Wade T.J., Cairney J. (2015). Sport participation and its association with social and psychological factors known to predict substance use and abuse among youth: A scoping review of the literature. Int. Rev. Sport Exerc. Psychol..

[17] Sommet N., Elliot A.J. (2017). Achievement goals, reasons for goal pursuit, and achievement goal complexes as predictors of beneficial outcomes: Is the influence of goals reducible to reasons?. J. Educ. Psychol..

[18] Lochbaum M., Kallinen V., Konttinen N. (2017). Task and ego goal orientations across the youth sports experience. Stud. Sport..

[19] Gjesdal S., Haug E.M., Ommundsen Y. (2019). A conditional process analysis of the coach-created mastery climate, task goal orientation, and competence satisfaction in youth soccer: The moderating role of controlling coach behavior. J. Appl. Sport Psychol..

[20] Ring C., Kavussanu M. (2018). The impact of achievement goals on cheating in sport. Psychol. Sport Exerc..

[21] Chamarro A., Carbonell X., Manresa J.M., Muñoz-Mirallles R., Ortega-González R., López-Morrón M.R., Batalla-Martínez C., Torán-Monserrat P. (2014). El Cuestionario de Experiencias Relacionadas con los Videojuegos (CERV): Un instrumento para detectar el uso problemático de videojuegos en adolescentes españoles. Adicciones.

[22] Mynard H., Joseph S. (2000). Development of the multidimensional peer-victimisation scale. Aggress. Behav..

[23] Cava M.J., Musitu G., Murgui S. (2007). Individual and social risk factors related to overt victimisation in a sample of Spanish adolescents. Psychol. Rep..

[24] Povedano A., Estévez E., Martínez B., Monreal M.C. (2012). A psychosocial profile of adolescent aggressors and school victims: Analysis of gender differences. Rev. Psicol. Soc..

[25] Little T.D., Henrich C.C., Jones S.M., Hawley P.H. (2003). Disentangling the “whys” from the “whats” of aggressive behaviour. Int. J. Behav. Dev..

[26] Estévez E. (2005). Violence, Victimization and School Rejection in Adolescence.

[27] Musitu G., Estévez E., Emler N. (2007). Adjustment problems in the family and school contexts, attitude towards authority and violent behaviour at school in adolescence. Adolescence.

[28] Newton M., Duda J.L., Yin Z. (2000). Examination of the psychometric properties of the Perceived Motivational Climate in Sport Questionnaire-2 in a sample of female athletes. J. Sport Sci..

[29] González-Cutre D., Sicilia A., Moreno J.A. (2008). Modelo cognitivo-social de la motivación de logro en educación física. Psicothema.

[30] Marsh H.W. (2007). Handbook of Sport Psychology.

[31] Brown C.F., Demaray M.K., Tennant J.E., Jenkins L.N. (2017). Cyber victimisation in high school: Measurement, overlap with face-to-face victimisation, and associations with social–emotional outcomes. Sch. Psychol. Rev..

[32] Castro-Sánchez M., Chacón-Cuberos R., Ubago-Jiménez J., Zafra-Santos E., Zurita-Ortega F. (2018). An explanatory model for the relationship between motivation in sport, victimization, and video game use in schoolchildren. Int. J. Environ. Res. Public Health.

[33] Chacón-Cuberos R., Castro-Sánchez M., González-Campos G., Zurita-Ortega F. (2018). Victimization in school, digital leisure and irritability: Analysis using structural equations. Relieve.

[34] Yang C., Sharkey J.D., Reed L.A., Chen C., Dowdy E. (2018). Bullying victimisation and student engagement in elementary, middle, and high schools: Moderating role of school climate. Sch. Psychol. Quart..

[35] Breske M.P., Fry M.D., Fry A.C., Hogue C.M. (2017). The effects of goal priming on cortisol responses in an ego-involving climate. Psychol. Sport Exerc..

[36] Kramer A.F., Colcombe S. (2018). Fitness effects on the cognitive function of older adults: A meta-analytic study. Perspect. Psychol. Sci..

[37] Arbour-Nicitopoulos K.P., Grassmann V., Orr K., McPherson A.C., Faulkner G.E., Wright F.V. (2018). A scoping review of inclusive out-of-school time physical activity programs for children and youth with physical disabilities. Adapt. Phys. Act. Q..

[38] Rhodes R.E., Lim C. (2018). Promoting parent and child physical activity together: Elicitation of potential intervention targets and preferences. Health Educ. Behav..

[39] Fontana M.S., Fry M.D., Cramer E. (2017). Exploring the relationship between athletes’ perceptions of the motivational climate to their compassion, self-compassion, shame, and pride in adult recreational sport. Measur. Phys. Educ. Exerc. Sci..

[40] O’Rourke D.J., Smith R.E., Punt S., Coppel D.B., Breiger D. (2017). Psychosocial correlates of young athletes’ self-reported concussion symptoms during the course of recovery. Sport Exerc. Perform. Psychol..

[41] Kenney E.L., Gortmaker S.L. (2017). United States adolescents’ television, computer, videogame, smartphone, and tablet use: Associations with sugary drinks, sleep, physical activity, and obesity. J. Pediat..

[42] Showell N.N., Cole K.W., Johnson K., DeCamp L.R., Bair-Merritt M., Thornton R.L. (2017). Neighbourhood and parental influences on diet and physical activity behaviors in young low-income paediatrics patients. Clin. Pediatr..

[43] Mazzone A., Nocentini A., Menesini E. (2017). Bullying and peer violence among children and adolescents in residential care settings: A review of the literature. Aggress. Violent Behav..

[44] Matthews C.R., Channon A. (2017). Understanding sports violence: Revisiting foundational explorations. Sport Soc..

[45] Graupensperger S.A., Jensen C.J., Evans M.B. (2018). A meta-analytic review of studies using the Prosocial and Antisocial Behavior in Sport Scale: Associations among intergroup moral behaviors. Sport Exerc. Perform. Psychol..

[46] Lodewyk K.R. (2018). Associations between university students’ personality traits and victimisation and its negative affect in school physical education. J. Phys. Educ. Sport.

[47] Gampell A.V., Gaillard J.C., Parsons M., Fisher K. (2017). Beyond stop disasters 2.0: An agenda for exploring the contribution of video games to learning about disasters. Environ. Hazards.

[48] Long J., Liu T., Liu Y., Hao W., Maurage P., Billieux J. (2018). Prevalence and correlates of problematic online gaming: A systematic review of the evidence published in chinese. Curr. Addict. Rep..

[49] Al-Yaaribi A., Kavussanu M. (2018). Consequences of prosocial and antisocial behaviors in adolescent male soccer players: The moderating role of motivational climate. Psychol. Sport Exerc..

[50] Martin E.M., Gould D., Ewing M.E. (2017). Youth’s perceptions of rule-breaking and antisocial behaviours: Gender, developmental level, and competitive level differences. Int. J. Sport Exer. Psychol..

[51] Bekiari A., Nikolaidou Z.A., Hasanagas N. (2017). Typology of motivation and aggression on the basis of social network variables: Examples of complementary and nested behavioral types through conventional statistics. Soc. Netw..

[52] Deliligka S., Bekiari A. (2018). Multi-parametric analysis of aggressive communication and motivation climate in physical education. Psychology.

[53] Shin H. (2017). Examining early adolescents’ peer climate using descriptive and status norms on academic engagement and aggressive behavior in the classroom. Asia Pac. Educ. Rev..

[54] Gabbiadini A., Riva P. (2018). The lone gamer: Social exclusion predicts violent video game preferences and fuels aggressive inclinations in adolescent players. Aggress. Behav..

[55] Kwak M., Oh I. (2017). Comparison of psychological and social characteristics among traditional, cyber, combined bullies, and non-involved. School Psychol. Int..

[56] Shams H., Garmaroudi G., Nedjat S. (2017). Factors related to bullying: A qualitative study of early adolescent students. Iran. Red Crescent Med. J..

[57] González J., Cayuela D., López-Mora C. (2019). Prosociality, physical education and emotional intelligence in school. J. Sport Health Res..

[58] Castro-Sánchez M., Ramírez-Granizo I.A. (2019). Application of psychomotor skills as a tool for social inclusion in early childhood education. ESHPA Educ. Sport Health Phys. Act..

[59] Massey W.V., Stellino M.B., Holliday M., Godbersen T., Rodia R., Kucher G., Wilkison M. (2017). The impact of a multi-component physical activity programme in low-income elementary schools. Health Educ. J..

